# Supplementary material for: Complex k-uniform tilings by a simple bitopic precursor self-assembled on Ag(001) surface
Source: Nat Commun. 2020 Apr 20;11:1856. doi: 10.1038/s41467-020-15727-6 (PMC7170884; doi:10.1038/s41467-020-15727-6)
Supplement: Supplementary file 1 — Supplementary Information [file 41467_2020_15727_MOESM1_ESM.pdf]

SUPPLEMENTARY INFORMATION

Complex *k*-Uniform Tilings from a Simple Bitopic Precursor Self-Assembled on Ag(001) Surface

Lukáš Kormoš et. al.

Supplementary Discussion

X-ray photoelectron spectroscopy analysis

The in-situ X-ray photoelectron spectroscopy analysis was performed for the 1U- to 3-U phases prepared in LEEM to ensure the purity and homogeneous coverage of each phase over the entire surface area. The molecular phase comprising pristine BDA was obtained directly after the deposition. In this way we can exclude the presence of minor phases which would influence the XPS conclusions. In particular, we exclude the presence of Ag adatoms. In addition to presented molecular phases we have identified several additional phases; some of them incorporating also Ag atoms, which can be clearly identified by STM. These phases show different geometrical arrangements and show a distinct diffraction patterns; hence they can be easily recognized in diffraction.

The XPS analysis was performed on a SPECS system equipped with a 150 mm mean radius hemispherical energy analyzer (Phoibos 150), a microchannel plate detector with a 2D CCD camera, and a conventional non-monochromatic Mg/Al X-ray source (XR 50). Mg Kα radiation with 300 W emission power (12.5 kV cathode-anode voltage) was used for all the measurements. A survey spectrum was measured in high magnification mode using a pass energy of 100 eV by integration of 5 sweeps with 0.1 s dwell time and 1 eV energy step. O 1s and C 1s (not shown) detailed spectra were acquired in high magnification mode using a pass energy of 20 eV integrating up to 180 sweeps with 0.1 s dwell time and 0.05 eV energy step. Normal emission geometry (emission angle 0°) was employed for all the measurements.

The Ag 3d peak was measured both before and after the measurement of the O 1s and C 1s peaks to ensure a correct binding energy reference during the prolonged measurements. No charge compensation or post measurement energy scale correction was employed. The measured energy position of the Ag $3d_{5/2}$ peak averaged for all the samples was (368.21 ± 0.01) eV at 0.95 confidence level for both the initial and final reference measurements. This value matches the recommended reference energy of (368.22 ± 0.01) eV for non-monochromatic Mg Kα radiation.[1] We observed a rigid shift of 0.1 – 0.2 eV of the O 1s and C 1s peaks between the initial 15 sweeps and all subsequent measurements. For consistency, we excluded these measurements from the averaging. The detailed spectra were fitted by 3 Voight components (the Lorentzian width of 0.5 eV) and the combined Shirley–linear background. The linear background component was

introduced to model a decreasing background intensity at the high binding energy side of Ag 3d peak.

LEEM analysis of molecular phases

We have derived the models presented in Figure 3 in the main text by analysis of diffraction patterns and STM images. By combination of diffraction (Supplementary Figures 1-3 b) and microdiffraction (Supplementary Figure 1-3 c) we have identified 4 distinct symmetry equivalent molecular domains. The real-space unit cell was determined from the associated reciprocal unit cells. Finally, we have calculated the diffraction patterns using LEEDpat software[2] (Supplementary Figures 1-3 e and f) and compared them to the measured ones (Supplementary Figures 1-3 d). As diffraction patterns are composed of 4 distinct domains with many diffraction spots, the mutual position of the individual spots presents a very precise way to confirm the validity of the prosed model. In Supplementary Figure 4 we show three distinct unit cells which show a good match with microdiffraction patters. Here, the mutual position of spots from distinct rotational domains in the marked areas enables to identify the correct unit cell. Moreover, this approach of 'local congruence' is insensitive to diffraction pattern distortions arising in the LEEM optical system.

The molecular arrangement in the model (Supplementary Figures 1-3 g) was derived combining the atomic resolution STM of the substrates and the STM images shown in Supplementary Figures 1-3 h. However, both LEED and STM do not provide exact position of molecules on the Ag(001) substrate. Here, we positioned the molecules in the way that the carboxylate moieties are in the on-top positions in line with the previous studied on related systems.[3,4] Summary of the derived models is given in Supplementary Figure 5.

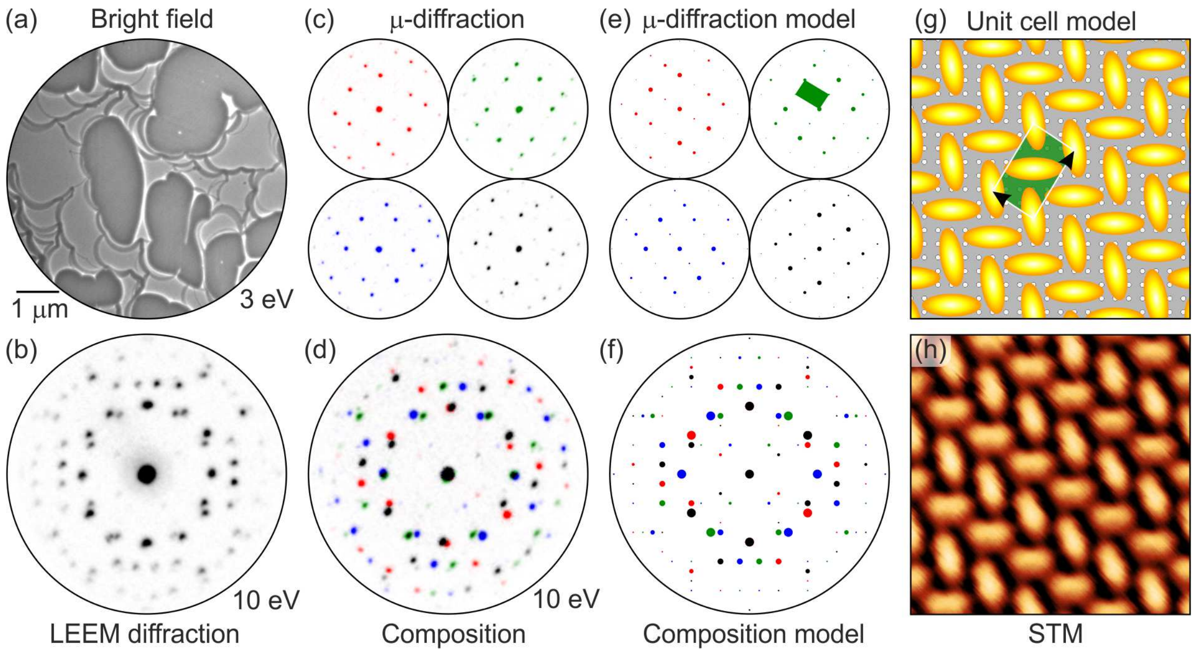

Supplementary Figure 1: LEEM and LEED analysis of the 1U-phase. (a) bright field LEEM image taken at 3 eV electron energy. Measured diffraction patterns at the electron energy of 10 eV: (b) area integrated diffraction, (c) μ-diffraction from circular are with diameter of 185 nm, and (d) composition of μ-diffraction patterns showing all spots of diffraction pattern given in (b). Theoretical diffraction patterns calculated from a model: (e) μ-diffraction patterns and (f) their composition. (g) Schematic model of molecular arrangement within the model and (h) its comparison with the STM image.

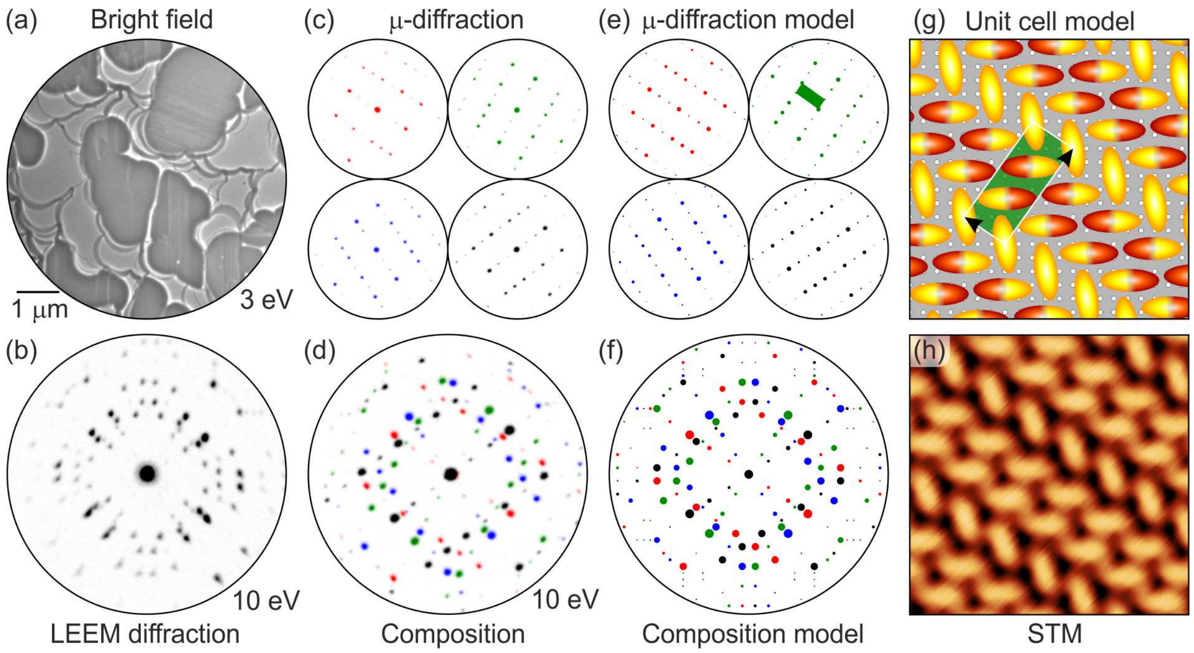

Supplementary Figure 2: Same as Supplementary Figure 1 but for the 2U phase.

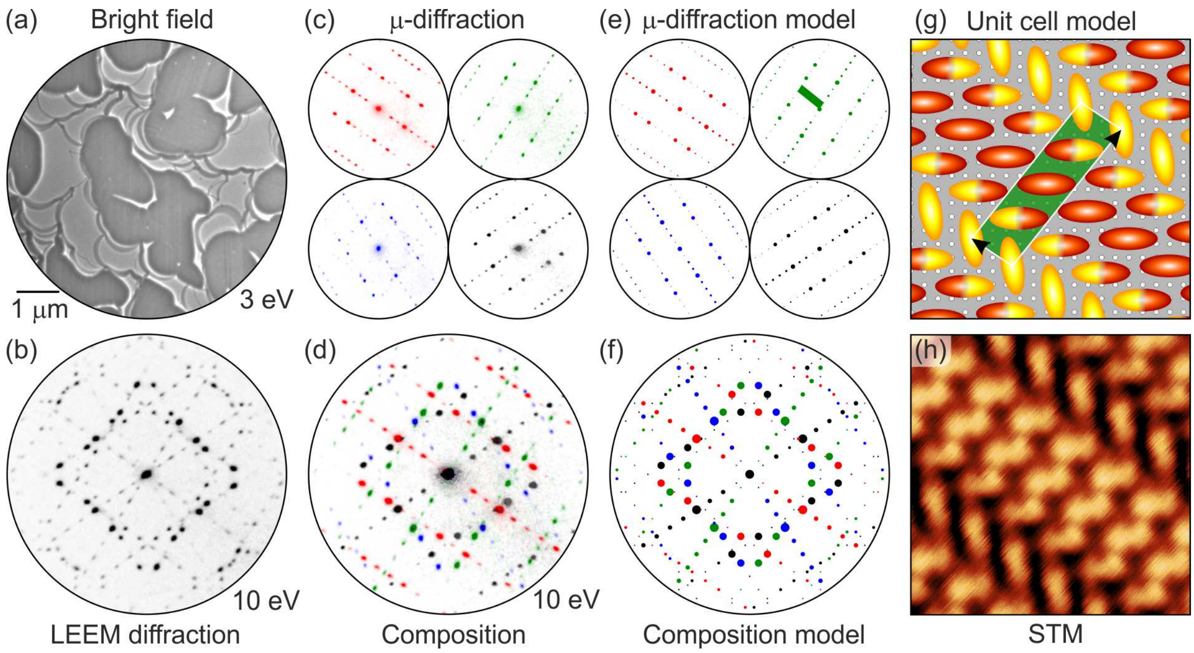

Supplementary Figure 3: Same as Supplementary Figure 1 but for the 3U phase.

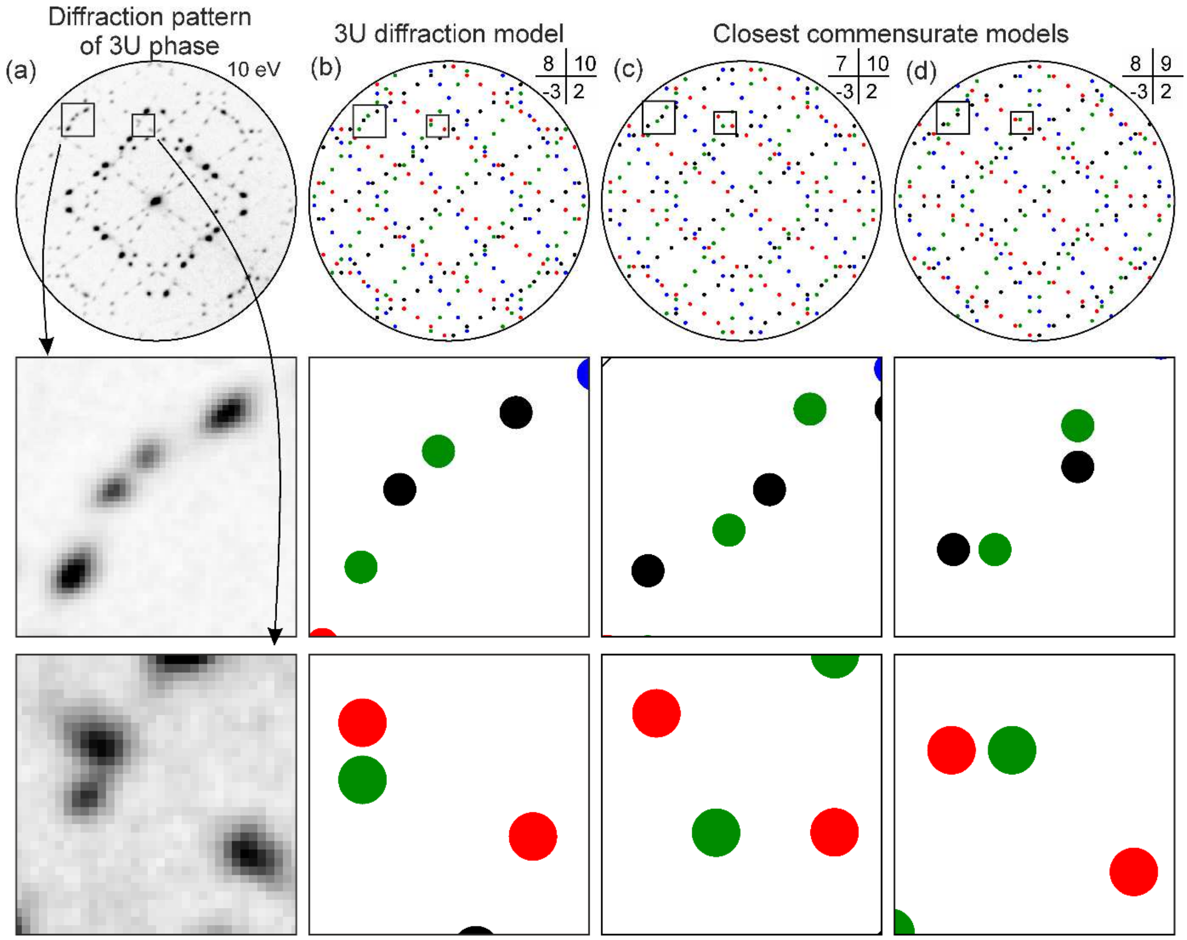

Supplementary Figure 4: (a) Measured diffraction pattern of 3U phase and (b-d) simulated diffraction patterns associated with proposed unit cells. Here the diffraction pattern for each of four rotational domains is presented in a different color. Whereas the simulated patterns for a single DBA domain are in a good match with measured one, the mutual position of diffraction spots from different domains provide a precise tool for identification of the unit cell that is closest the experimental one. In the bottom panels the selected parts of diffraction pattern are enlarged to enable an easy comparison of the local patterns. The closes match is observed for model given in (b).

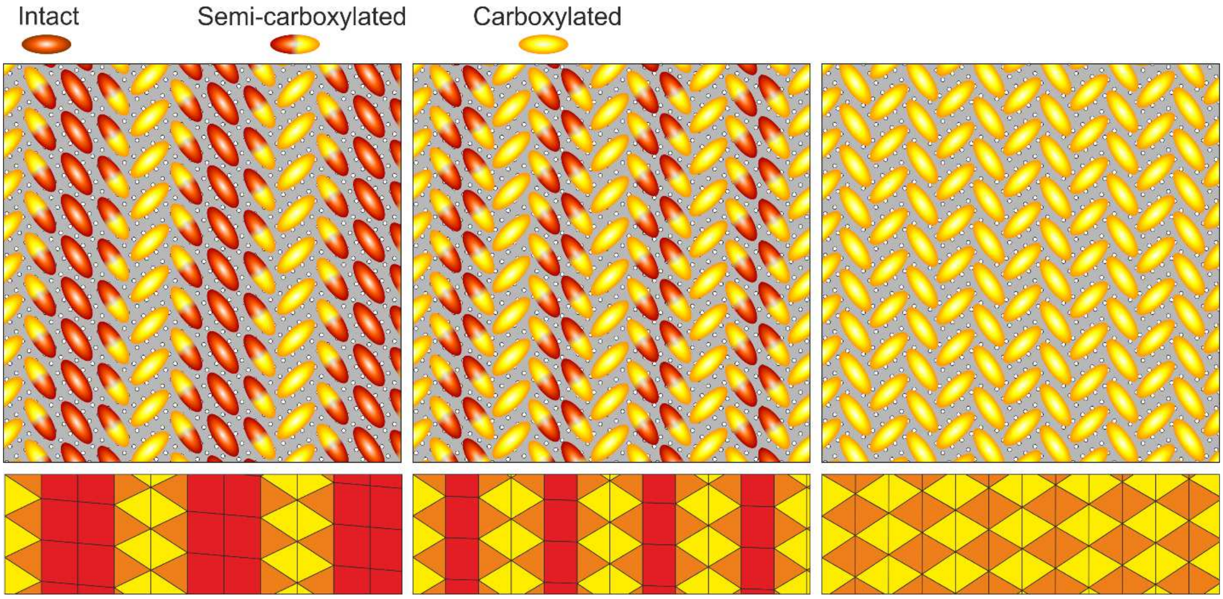

Supplementary Figure 5: (a-c) Summary of models of 3U, 2U and 1U phases and (d-f) the associated surface tiling. The chemical state of carboxyl/carboxylate moieties is depicted by red/yellow color on the particular half of the BDA represented by the ovals.

Long range order over large areas

The presence of the long-range order of the described phases is supported by the clear diffraction patterns given in Supplementary Figures 1, 2, and 3. Typically, the diffraction images in LEEM are acquired over larger areas of approx. 150 μm^2 and μ-diffraction from circular areas with diameter of 185 nm. Patterns were recorded on multiple sample positions.

Additionally, long range order of the 1U and 2U phases was observed in the STM images as shown in the Supplementary Figures 6a and b. The 3U phase slowly transforms during the sample transfer and prolonged STM measurements, therefore as shown in the Supplementary Figure 6c, the 3U phase usually coexist on the surface with the mixed phase presented in Supplementary Figure 8.

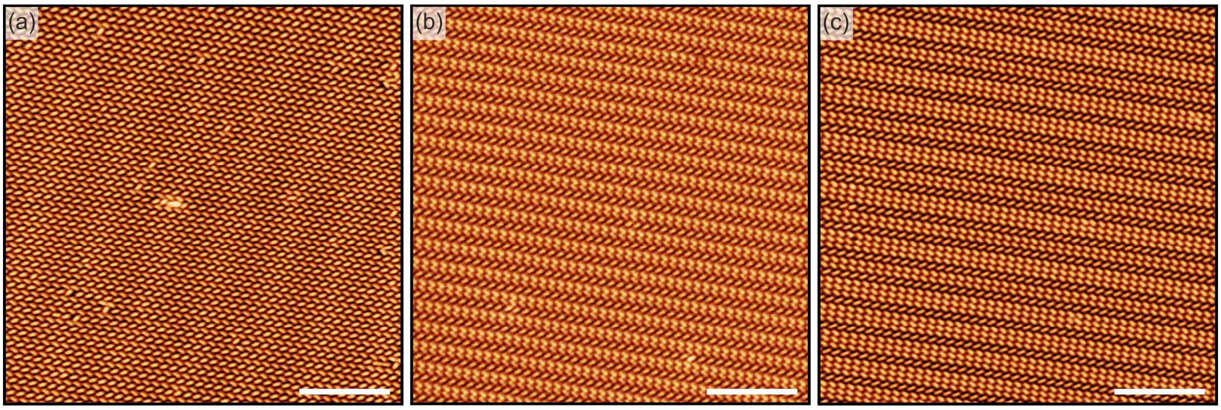

Supplementary Figure 6: STM images of (a) 1U and (b) 2U and (c) 3U phases. In (c) the 3U phase (bottom) coexist with the mixed phase (top). Scale bars are 10 nm; no thermal drift correction was applied in these images.

Sequence of 2U- and 3U-phases

The deprotonation of the BDA slowly continues even at the room temperature and a new mixed phase forms on the surface with ratio of 2H-, 1H-, and 0H-BDA distinct from the pure 3U and 2U phases. Consequently, the 3U phase internally reassembles to the 2U phase. Both 3U and 2U phases can seamlessly interconnect as the position and orientation of 0H-BDA molecules in respect to substrate is the same for both phases: they possess the same unit cell vector $-3\vec{a}_1 + 2\vec{a}_2$ along the yellow marked connecting line in Supplementary Figure 7.

As stripes of both 2U and 3U phases are commensurate with the substrate and match together without implying any structural distortion we infer that the sequence of the 2U and 3U stipes is random. Additionally, as shown in Supplementary Figure 8 some areas display four 2H-BDA molecules in a row. These represent 4-Uniform tiling featuring 3 distinct vertices. In contrast to the 3U and 2U phases, these are, however, observed only as local inclusions in the mixed phase.

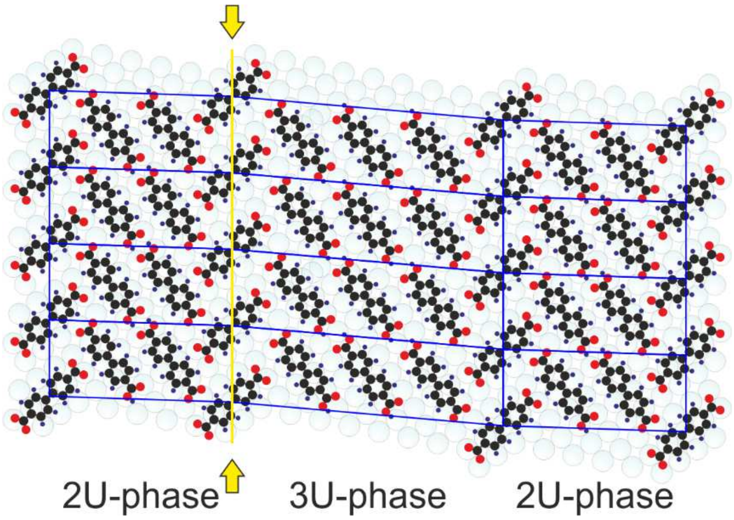

Supplementary Figure 7: Extended molecular structure of intermediate phase joining 3U- and 2-U phases. Line along which unit cells and structure of 2U- and 3U-phases matches is highlighted by yellow line marked by arrows.

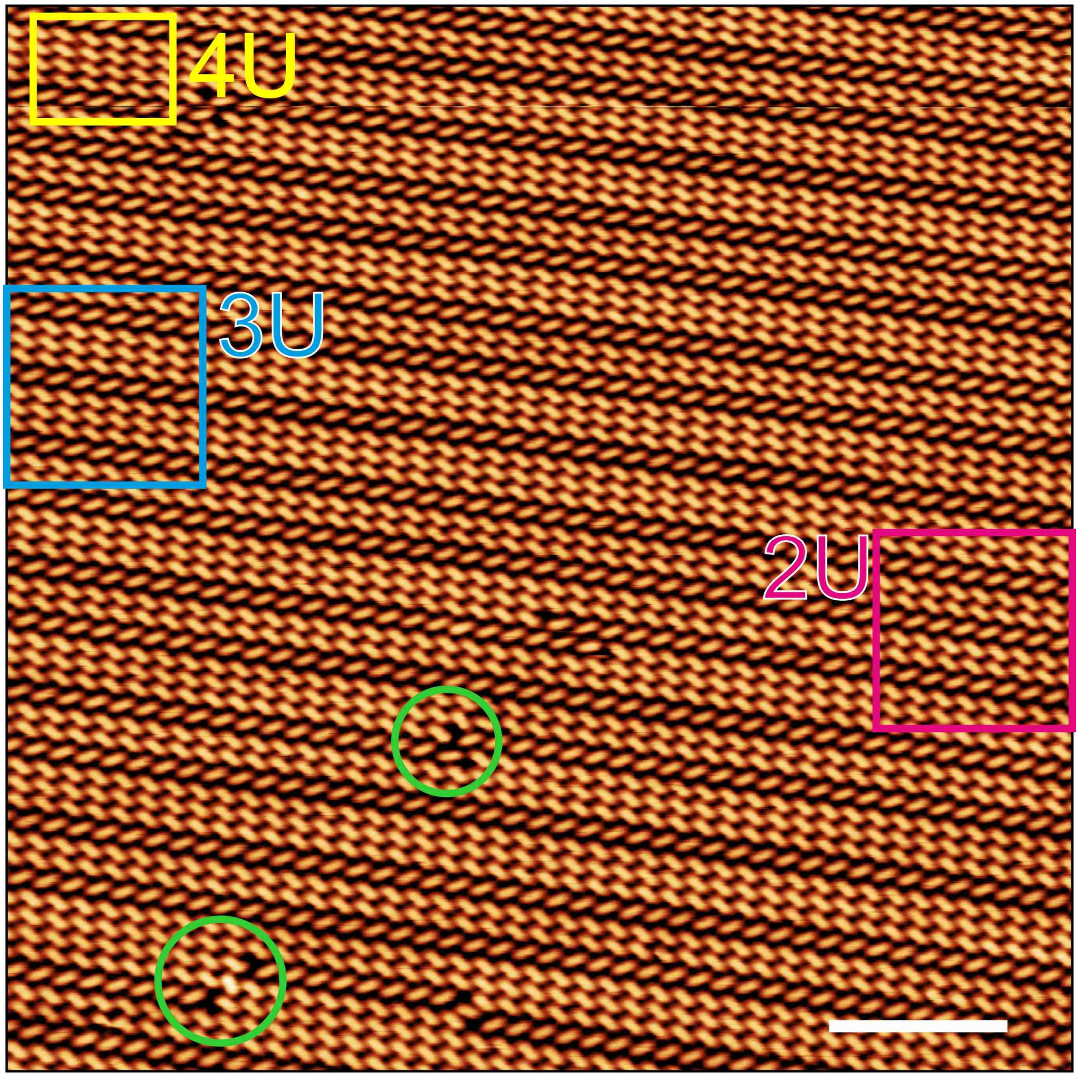

Supplementary Figure 8. STM image of a mixed phase on Ag(001) featuring the stripes 2U and 3U phases. Cyan and magenta squares mark 3U and 2U parts, respectively. Green Circles highlights parts in which the phase transformation occurs, and yellow square mark local inclusions of 4-Uniform tilings. Scale bar is 5 nm; acquisition parameters: -350 mV, 50 pA; no thermal drift correction.

Phase transformations

The phase transformation between 3U and 2U phases was followed by LEEM in the bright field mode. Sequence of images cut from the Supplementary Movie 1 is shown in Supplementary Figure 9. During the annealing at elevated temperatures a gradual BDA deprotonation occurs. Consequently, we observe the transformation of associated molecular phases within the larger islands. Both 3U and 2U phases show similar stripe structure differing only in the stripe width: there is an extra 2H-BDA in between two 1H-BDA in 2U phase compared to 2U as shown in Figure 4 in the main text. Moreover, these two phases can be seamlessly connected as demonstrated in Supplementary Discussion, Sequence of 2U- and 3U-phases. The 3U → 2U phase transformation takes place within the 3U islands along phase boundary propagating along the molecular stripes; the rate of transformation is further enhanced by voids present in otherwise compact molecular phase. We infer that the seamless connection is the key in enabling the internal transformation mechanism as a sharp phase boundary may exist.

The phase transformation starts from the periphery of the molecular islands or from a larger void within the 3U islands as demonstrated in Supplementary Figure 9. Supplementary Figure 9a shows the first transformation that starts from the island periphery: the areas marked by the arrows are quickly transformed to 2U phase. However, only a part of the island is transformed: the boundary of 3U and 2U phases marked by the arrow in Supplementary Figure 9b is advancing slowly in comparison with quite fast initial transformation. When the boundary reaches a void already present within the island, the phase boundary starts to move very quickly: the 2U phase is grows at the expense of consumed 3U phase at the opposite side of the void. The other parts of the islands are subsequently transformed following this mechanism. The 2U → 1U transformation proceed in the similar way.

Since the presence of the voids largely enhances the transformation rate, we infer that these play and important role in the phase transformation: the molecular rearrangement requires an open surface area to proceed without a large activation barrier associated with BDA detachment from surface. The void mediation therefore enhances transformation rate as all molecules remain in contact with surface. The phase transformation can also proceed near the defect in the molecular layer as marked by green circles in the STM image shown in Supplementary Figure 8. However,

we expect that this transformation is much slower as there is very limited possibility of molecular rearrangement.

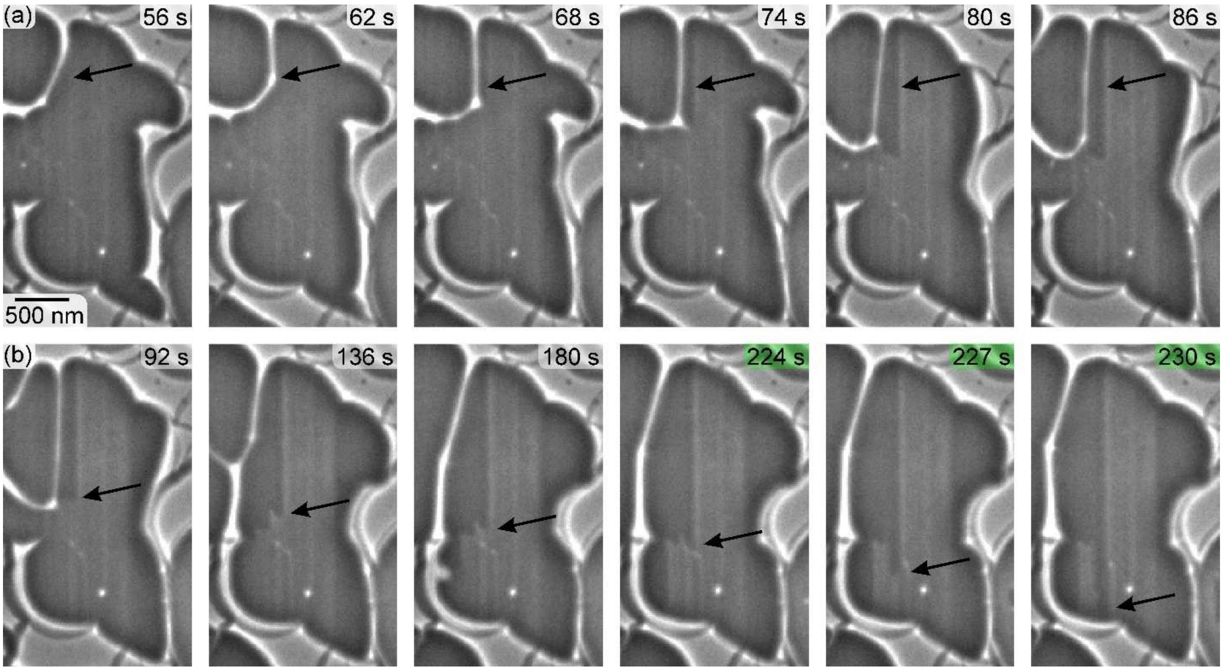

Supplementary Figure 9: Image sequence taken from the real-time LEEM video (Supplementary Movie 1) recorded during the 3U → 2U phase transformation: (a) initial fast transformation, (b) slow advancement. The video was taken in the bright field mode at 1.5 eV. The sample temperature was gradually increasing from 420 to 425 K. In the top row the fast growth of 2U phase (a darker area) from the side of the 3U phase island (light gray areas) as marked by black arrow. The bottom line shows the slow progress of 2U phase (marked by the black arrow) until a void in the island is reached; then a rapid progress of the phase boundary is observed. The time information in top right corner is time from the start of Supplementary Movie 1.

Supplementary References

1. Seah, M. P., Gilmore, I. S. & Beamson, G. XPS: binding energy calibration of electron spectrometers 5:re-evaluation of the reference energies. *Surf. Interface Anal.* **26**, 642–649 (1998).
2. LEEDpat, Version 4.2, utility by K.E. Hermann (FHI) and M.A. Van Hove (HKBU), Berlin / Hong Kong, 2014; see also http://www.fhi-berlin.mpg.de/KHsoftware/LEEDpat/index.html
3. Schmitt, T., Hammer, L. & Schneider, M. A. Evidence for On-Site Carboxylation in the Self-Assembly of 4,4 ′ - Biphenyl Dicarboxylic Acid on Cu(111). *J. Phys. Chem. C* **120**, 1043–1048 (2016).
4. Fuhr, J. D. *et al.* Interplay between Hydrogen Bonding and Molecule − Substrate Interactions in the Case of Terephthalic Acid Molecules on Cu(001) Surfaces. *J. Phys. Chem. C* **117**, 1287–1296 (2013).
