# Supplementary material for: Complex k-uniform tilings by a simple bitopic precursor self-assembled on Ag(001) surface
Source: Nat Commun. 2020 Apr 20;11:1856. doi: 10.1038/s41467-020-15727-6 (PMC7170884; doi:10.1038/s41467-020-15727-6)
Supplement: Supplementary file 3 — Description of Additional Supplementary Files [file 41467_2020_15727_MOESM3_ESM.pdf]

Description of Additional Supplementary Files

File Name: Supplementary Movie 1
Description: Real-time LEEM video recorded during the 3U → 2U phase transformation. The video was taken in the bright field mode at 1.5 eV while the sample temperature was gradually increasing from 420 to 425 K. White rectangle marks the area shown in Supplementary Figure 9.
